# Attenuation of Vanadium-Induced Neurotoxicity in Rat Hippocampal Slices (In Vitro) and Mice (In Vivo) by ZA-II-05, a Novel NMDA-Receptor Antagonist

**DOI:** 10.3390/ijms242316710

**Published:** 2023-11-24

**Authors:** Amany Digal Ladagu, Funmilayo Eniola Olopade, Paul Chazot, Ademola A. Oyagbemi, Samuel Ohiomokhare, Oluwabusayo Racheal Folarin, Taidinda Tashara Gilbert, Madison Fuller, Toan Luong, Adeboye Adejare, James O. Olopade

**Affiliations:** 1 Department of Veterinary Anatomy, University of Ibadan, Ibadan 200284, Nigeria; amanykladagu@ymail.com (A.D.L.); oluwabusayoracheal@gmail.com (O.R.F.); giberttashara@gmail.com (T.T.G.); jkayodeolopade@yahoo.com (J.O.O.); 2 Department of Anatomy, College of Medicine, University of Ibadan, Ibadan 200284, Nigeria; funmiolopade@yahoo.com; 3 Department of Biosciences, Durham University, County Durham DH1 3LE, UK; samokhare@yahoo.com; 4 Department of Veterinary Physiology and Biochemistry, Faculty of Veterinary Medicine, University of Ibadan, Ibadan 200284, Nigeria; ademola.oyagbemi778@gmail.com; 5 Department of Neuroscience, College of Arts and Sciences, Saint Joseph’s University, Philadelphia, PA 19131, USA; fmadison78@gmail.com (M.F.); toan.luong@sju.edu (T.L.); 6 Department of Pharmaceutical Sciences, Philadelphia College of Pharmacy, Saint Joseph’s University, Philadelphia, PA 19131, USA; aadejare@sju.edu

**Keywords:** Alzheimer’s disease, vanadium, NMDA-receptor antagonist, neurotoxicity, hippocampus

## Abstract

Exposure to heavy metals, such as vanadium, poses an ongoing environmental and health threat, heightening the risk of neurodegenerative disorders. While several compounds have shown promise in mitigating vanadium toxicity, their efficacy is limited. Effective strategies involve targeting specific subunits of the NMDA receptor, a glutamate receptor linked to neurodegenerative conditions. The potential neuroprotective effects of ZA-II-05, an NMDA receptor antagonist, against vanadium-induced neurotoxicity were explored in this study. Organotypic rat hippocampal slices, and live mice, were used as models to comprehensively evaluate the compound’s impact. Targeted in vivo fluorescence analyses of the hippocampal slices using propidium iodide as a marker for cell death was utilized. The in vivo study involved five dams, each with eight pups, which were randomly assigned to five experimental groups (*n* = 8 pups). After administering treatments intraperitoneally over six months, various brain regions were assessed for neuropathologies using different immunohistochemical markers. High fluorescence intensity was observed in the hippocampal slices treated with vanadium, signifying cell death. Vanadium-exposed mice exhibited demyelination, microgliosis, and neuronal cell loss. Significantly, treatment with ZA-II-05 resulted in reduced cellular death in the rat hippocampal slices and preserved cellular integrity and morphological architecture in different anatomical regions, suggesting its potential in countering vanadium-induced neurotoxicity.

## 1. Introduction

Vanadium, an element with the atomic number 23, belongs to the transition series and finds extensive use in the chemical industry [1]. A significant source of vanadium toxicity is the combustion of fossil fuels, as observed in regions such as the Gulf of Mexico [2], the Nigerian Niger Delta area [3] and the Arabian Gulf [4]. Importantly, vanadium has the ability to cross the blood–brain barrier [5]. Studies that involved administering vanadium to experimental animals have reported various detrimental effects, including demyelination, activation of microglia and astrocytes, increased expression of tumor necrosis factor and interleukin 1β, as well as cognition and locomotion deficits [6]. Experimental studies in mice and rats have demonstrated that exposure to vanadium during lactation results in behavioral deficits; damage to oligodendrocytes, leading to hypomyelination; and BBB impairment [7,8]. Activated astrocytes have also been observed in neonatal rats [9] and suckling mice following vanadium exposure. Inhalation of vanadium pentoxide by adult mice leads to damage to hippocampal neurons and memory impairment [10], as well as damage to ependymal cells [11] and olfactory impairment [12]. Chronic exposure to vanadium in adults has also been associated with neurotoxic effects. Occupational exposure to vanadium has been linked to cognitive alterations in humans [13]. It remains a significant concern and continues to impact various ecosystems and organisms.

Several compounds have been proposed for treating vanadium neurotoxicity in vivo. Erythropoietin [7], kolaviron [14], and vitamin E have all shown promise in this regard [2,15]. However, these compounds have exhibited limitations, such as severe pro-oxidative effects at the required dose for treatment of vanadium; since vanadium exposure is a topical and ongoing occurrence, the development and testing of newer antidotes remains pertinent. Extensive research has highlighted the significance of Sigma receptors, Dopamine receptors, Opioid receptors and N-methyl-D-aspartate (NMDA) receptors, a family of glutamate receptors, in various neurological and neurodegenerative diseases, including Alzheimer’s disease (AD) [16]. These receptors play a crucial role in processes such as learning, memory acquisition, brain plasticity, neuronal survival, and overall brain development. Their activation is essential for normal physiological functions in the central nervous system (CNS) [17]. Calcium ion influx mediated by NMDA receptors is particularly important for memory, synaptic transmission, synaptic plasticity, and the development of the nervous system. However, excessive NMDA receptor stimulation and calcium ions influx can lead to excitotoxicity and neuronal death in pathological conditions [18]. Targeting specific subunits of NMDA receptors, particularly GluN2A and GluN2B, through selective antagonism, has proven to be an effective approach in mitigating the pathological effects of excitotoxicity associated with the progression of neurodegenerative diseases like AD, Parkinson’s disease (PD), depression, and neuropathic pain [19,20].

In this study, our main objective was to investigate the neuroprotective and beneficial effects of ZA-II-05, also known as Compound C, a novel NMDA antagonist and multi-targeted directed ligand, in mitigating vanadium-induced neurotoxicity. We utilized two model systems, including rat hippocampal slices and mice, to comprehensively examine the effects of ZA-II-05. The findings from this study hold significant potential in identifying promising candidate(s) for clinical trials and therapeutic interventions against neurodegenerative diseases. The translation of these findings into clinical practice could eventually alleviate the societal burden associated with the care of patients suffering from neurological disorders. 

## 2. Results

### 2.1. Calcium Mobilisation on Vanadium-Treated CAD Cells

**Figure 1:**
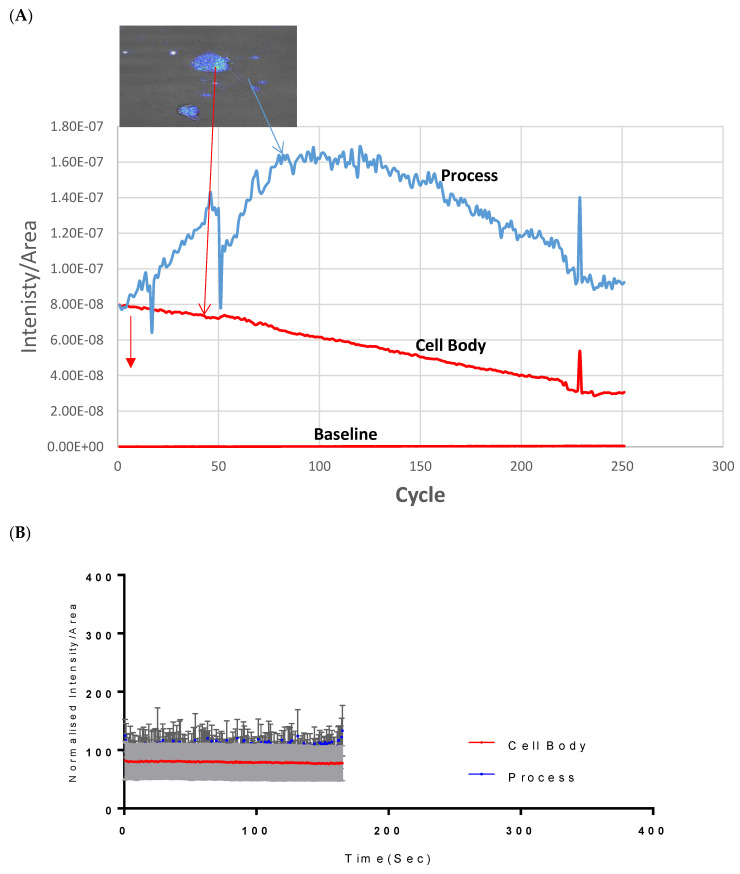

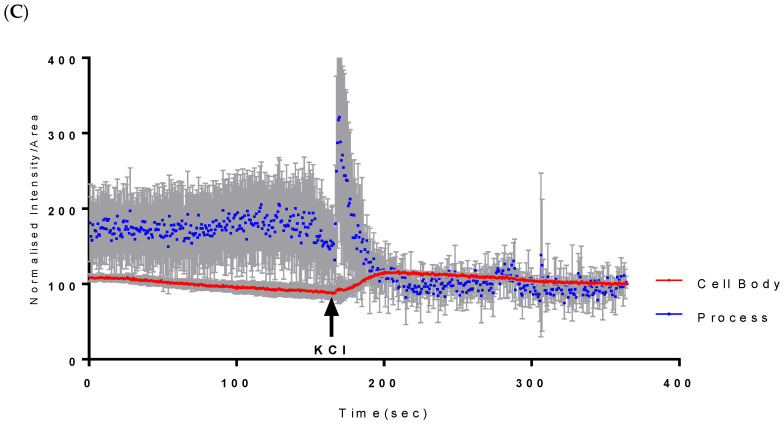
Effects of vanadium on calcium mobilization in differentiated CAD cells. (**A**) Vanadium, (**B**) control, (**C**) KCl. Vanadium (500 µM) was applied (red arrow) and recorded for 250 cycles. Time course of calcium responses in individual Fura-2 AM loaded neuronal CAD cells is indicated by colored lines. Following a 2 min baseline period, perfusion for 1 min with 500 µM vanadium elicited a large calcium response in the process, with little response in the cell body. Both responded to depolarization with 60 mM KCl, indicating electrically excitable neurons.

Assessment of both the processes and the cell body of CAD cells showed that at 500 µM, Vanadium elicited a rapid calcium response, especially compared to that of the neuronal cell body, which peaked and dropped steadily to the original level starting concentration at cycle 250, as seen in Figure 1A. Little or no response was observed in the cell body in CAD cells. Figure 1B,C shows the status of calcium ions in the control group and after depolarization with KCl, respectively.

### 2.2. Binding Profile of ZA-II-05 on Neurotransmitters and Transporters

**Figure 2:**
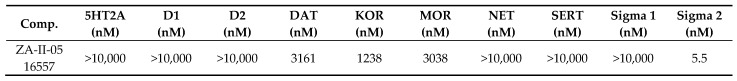

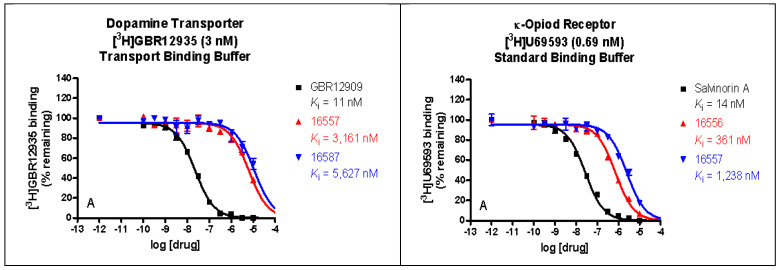

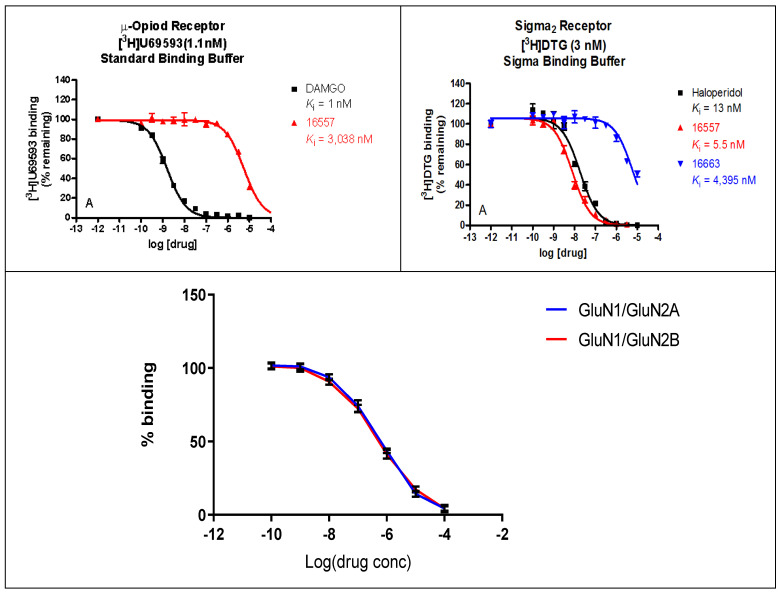
In vitro binding affinities of novel ZA-II-05 for 5-HT2A, D1, D2, DAT, KOR, MOR, NET, SERT, Sigma-1, and Sigma-2 receptors. The IC_50_ and Ki for ZA-II-05 subjected to further analysis and (+)-MK-801 run under experimental condition as reference. Expression of subtype ligands in transfected HEK Cells; [^3^H] MK801 binding assay (n = 3 individual assays) using transfected HEK293 cells (1:3 cDNA ratio) for GluN1: GluN2). No significant difference in affinity for GluN1/GluN2A and GluN1/GluN2B (Mean IC_50_ (M) GluN1/GuN2A: 5.178 × 10^−7^ M and GluN1/GluN2B: 4.289 × 10^−7^ M).

The binding affinities of ZA-II-05 were highest at the sigma 2, KOR, MOR, and DAT receptors, compared to 5HT2A, D1, D2, NET, SERT, and Sigma 1, which had lower binding affinities of over 10,000 nM as seen in Figure 2. GluN 2A and GluN 2B were observed to have affinities within close ranges with a mean IC_50_ of 5.178 × 10^−7^ M and 4.289 × 10^−7^ M, respectively.

### 2.3. Biochemical Studies

**Figure 3:**
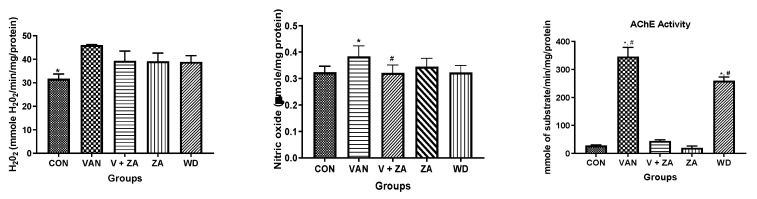

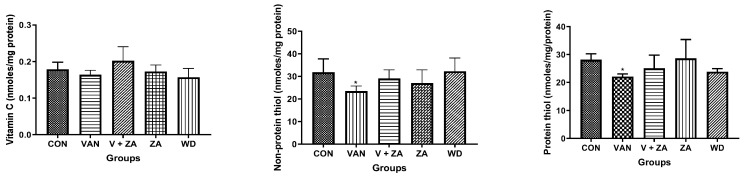
Effect of vanadium-induced neurotoxicity and the reversal effect of ZA-II-05 on hydrogen peroxide, (H_2_O_2_) generation, nitric oxide (NO) content, acetylcholinesterase activity and non-enzymatic antioxidant content in mice brain. The symbol * is used to represent a significant difference compared to the control group at a significance level of *p* < 0.05, while the symbol # indicates a significant difference compared to vanadium + ZA-II-05 (V+ZA) group at the same significance level. Vanadium (3 mg/kg) and ZA-II-05 (100 mg/kg). CON, control; VAN, vanadium; ZA; ZA-II-05; WD, withdrawal.

Evaluation of neuroprotective effects of ZA-II-05 using different biochemical markers (H_2_O_2_, NO content, protein thiol and non-protein thiol) showed high significance in the activity of AChe compared to other biochemical markers, as seen in Figure 3. The Ache content was significantly (*p* < 0.05) reduced in the control and compound-treated groups compared to the vanadium and withdrawal groups.

### 2.4. In Vitro Neuroprotection from Vanadium Toxicity Hippocampal Slices

**Figure 4:**
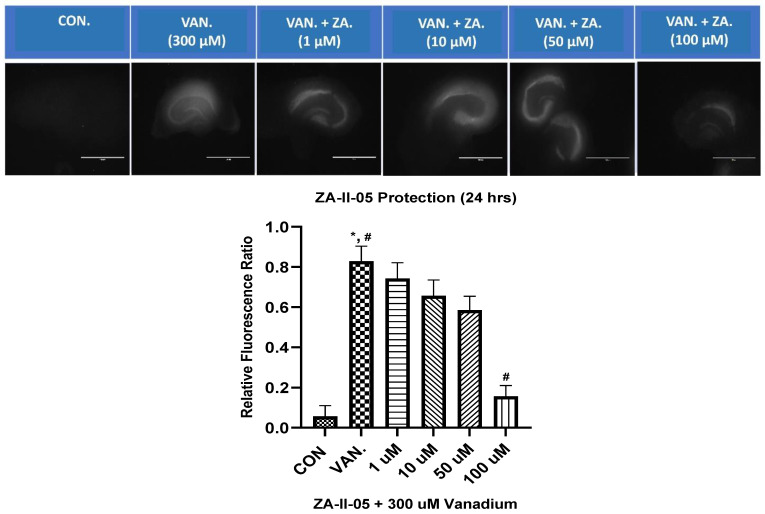
In vitro hippocampal slice cultures showing neuroprotection of ZA-II-05 at different concentrations (1 µM, 10 µM, 50 µM, 100 µM) against vanadium-induced neurotoxicity in rat brain hippocampal slices. Propidium iodide fluorescence is proportional to cell death. Results for hippocampal slice neuroprotection from 300 µM vanadium induced toxicity are presented as measured via propidium iodide uptake. t0 initial image (1), t24 h treatment (2) and tGLUT (3) following 50 mM glutamate. Protection against vanadium was significant at 100 μM (*p* < 0.05). Data are shown as mean ± S.E.M from n = 15–18 hippocampal slices per group. ImageJ manual selection feature was used for assessment using basic pixel intensity measurement in all the images to determine the relative fluorescence ratio using the equation: (t4 (or t24) − t0/tGLUT − t0). The symbol * is used to represent a significant difference compared to the control group at a significance level of *p* < 0.05, while the symbol # indicates a significant difference compared to vanadium group at the same significance level. Scale bar: 100 μm remains consistent across the rows.

ZA-II-05 was evaluated for its neuroprotective effect against vanadium-induced neurotoxicity at 300 µM. This was performed at different concentrations of ZA-II-O5, as seen in Figure 4. There was a gradual reduction in cell death with increasing concentrations of the compound. Peak protection was achieved at 100 µM at a significant level (*p* < 0.05) compared to the vanadium (300 µM)-treated hippocampal slices.

### 2.5. Histopathology 

#### 2.5.1. Expression of Neuronal Count/Density (NeuN) in Different Regions of the Brain Treated with Vanadium and ZA-II-05 

**Figure 5:**
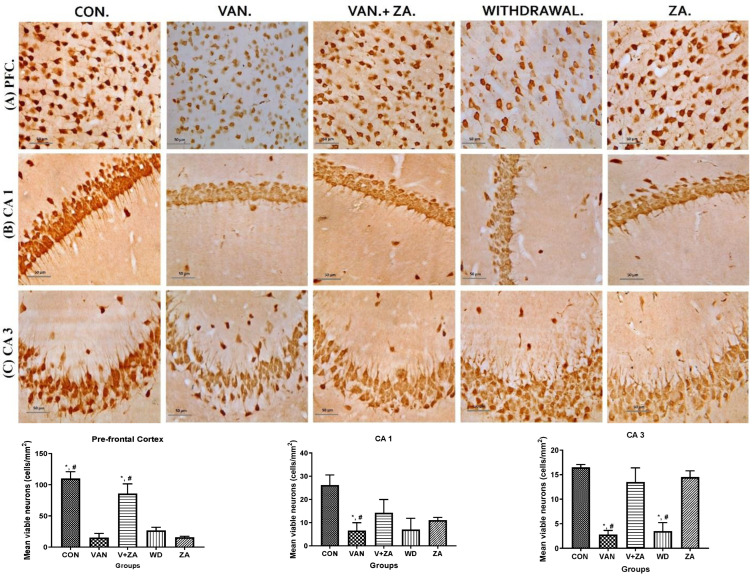
Presents the results obtained from NeuN immunostaining of the prefrontal cortex (PFC), CA 1, and CA 3 hippocampal regions; (**A**) PFC reveals neurons with no significant abnormalities observed in the cortical sections of the control group. Normal neurons were characterized by rounded and pale nuclei, while degenerating neurons displayed smaller cell bodies with neuropil vacuolation and significantly higher pyknotic nuclei compared to the control group. The ZA-II-05 group exhibited significantly less cellular pathology in the prefrontal cortex compared to the vanadium-exposed groups, indicating a potent protective effect against vanadium-induced damage; (**B**) CA1 region shows progressive loss of apical dendrites in the pyramidal cells of the dorsal hippocampus after vanadium treatment. ZA-II-05-treated group displayed a reversal effect relative to the vanadium-exposed group, suggesting a mitigation of the dendritic loss; (**C**) CA3 region revealed neuronal degeneration in the pyramidal cells after vanadium exposure. The vanadium-treated group exhibited morphological alterations in cortical pyramidal cells, including cytoplasmic vacuolation, cell clustering, pyknosis, and loss of layering pattern, compared to the control and ZA-II-05 treated groups, which displayed normal neuronal morphology. The ZA-II-05-treated group showed a reversal effect with reduced cellular toxicity. Interestingly, ZA-II-05-treated group decreased viable cells in CA1 but increased neuronal number and vitality in the prefrontal cortex. The symbol * indicates a significant difference compared to the control group at *p* < 0.05, while the symbol # indicates a significant difference compared to the vanadium + ZA-II-05 (V+ZA) group at *p* < 0.05. Vanadium was administered at a dose of 100 mg/kg, and ZA-II-05 was administered at a dose of 100 mg/kg. Abbreviations: CON for control, VAN for vanadium, ZA for ZA-II-05, and WD for withdrawal. Magnification: ×400. Scale bar: 50 μm remains consistent across the rows.

Different brain regions were examined to determine their neuronal vitality and number, and it was revealed that vanadium treatment caused a significant reduction in neuronal count and high levels of pyknosis. A higher number of viable neuronal cells was observed in the control, Van + ZA-, and ZA-treated groups. The nuclei in the vanadium-treated group appear to be pale with high cytoplasmic vacuolations, which was significantly lower (*p* < 0.05), as seen in all three anatomic regions (PFC, CA1 and CA3) in Figure 5. Interestingly, ZA treatment only seems to show an increased number of neurons in the PFC with high vitality. 

#### 2.5.2. Quantification of Dendritic Density and Arborizations

**Figure 6:**
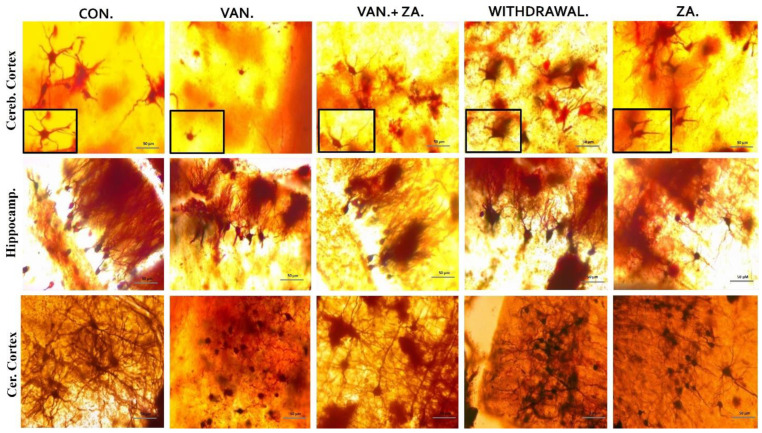
Golgi stain showing the soma of neurons and their apical dendrites. Note the shortened dendrites and alterations in the granule cell of vanadium treated groups, compared with control and ZA-II-05-treated groups. Little or no apical and basal dendrites were observed with less arborization. ZA-II-05 coadministration reversed dendritic changes due to vanadium neurotoxicity. There was increased dendritic length and arborization compared to the vanadium-treated group. Magnification ×40 (insert magnification ×100). Scale bar: 50 μm remains consistent across the rows.

In our observations, we noted significant reductions in dendritic length and alterations in the granule cells of the vanadium-treated groups. Specifically, we observed smaller, sparser, and less elaborate dendritic arborizations, with limited or absent apical and basal dendrites in the hippocampus, cerebral, and cerebellar regions of the mice brains (Figure 6). Coadministration of ZA-II-05 successfully reversed these dendritic changes induced by vanadium neurotoxicity by a significant amount. Notably, the treatment resulted in increased dendritic lengthening and branching and enhanced arborization compared to the vanadium-treated group. 

#### 2.5.3. Expression of Myelin Basic Protein (MBP) in Different Regions of the Brain Treated with Vanadium and ZA-II-05

**Figure 7:**
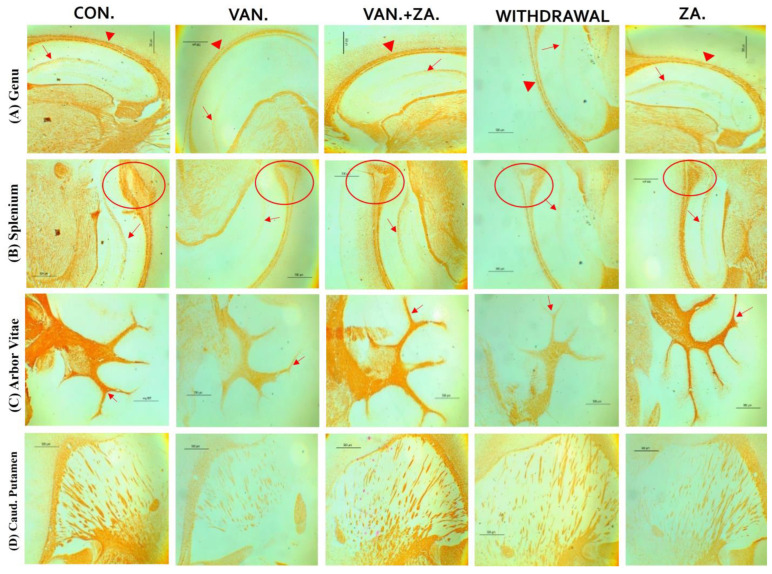

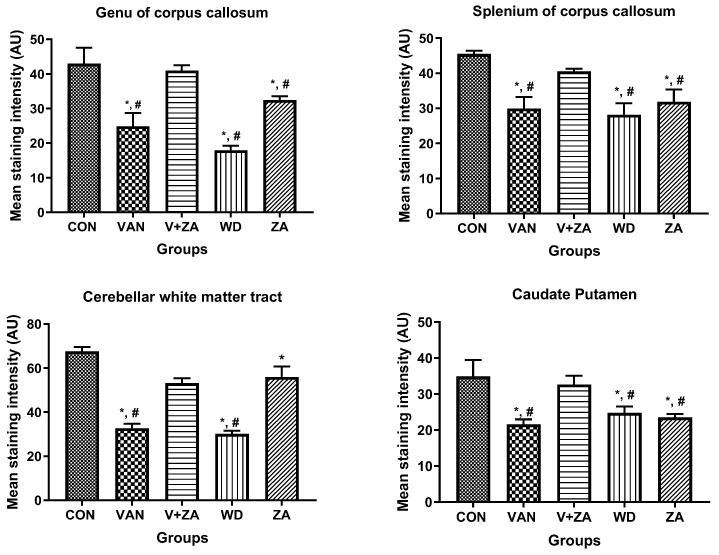
Panel A presents a micrograph of the whole brain stained with Myelin Basic Protein (MBP). The labeled regions include the genu (**A**), splenium (**B**) of the corpus callosum, arbor vitae (**C**), and the caudate putamen (**D**), which were the main areas analyzed for myelination. The control group exhibited closely arranged and uncluttered myelin fibers. However, in the brains of vanadium-treated mice, clear myelin damage was observed compared to the control group. The myelin pallor showed homogeneous depletion, indicating myelin damage caused by lipid peroxidation. Demyelination, with a reduced density of myelin tracts, was evident in fibers (**A**–**D**). Conversely, coadministration of ZA-II-05 and vanadium resulted in closely and orderly arranged myelin fibers. In the ZA-II-05 treated vanadium group, myelin recovery and preservation of axonal dendritic integrity in the deep cortical layers were observed. In the statistical analysis, the symbol * denotes a significant difference compared to the control at *p* < 0.05, while # indicates a significant difference compared to the vanadium + ZA-II-05 (V+ZA) group at *p* < 0.05. Vanadium was administered at a dose of 100 mg/kg, and ZA-II-05 at a dose of 100 mg/kg. Abbreviations used are as follows: CON for the control group, VAN for the vanadium-treated group, ZA for the ZA-II-05-treated group, and WD for the withdrawal group. Scale bar: 50 μm remains consistent across the rows.

Histopathological examination of vanadium-exposed brains exhibited evident myelin damage compared to the control group. Demyelination was observed in the fibers of the genu, splenium, arbor vitae, and the caudate putamen regions of mice brains, accompanied by a reduced density of myelin tracts. The myelin pallor appeared uniformly depleted, as seen in Figure 7. Control and ZA-II-05-treated groups showed significantly higher expression of myelin content and preservation of axonal dendritic integrity in the deep cortical layers (*p* < 0.05) compared to the vanadium-treated group.

#### 2.5.4. Expression of Glial Fibrillary Protein (GFAP)in Different Regions of the Brain Treated with Vanadium and ZA-II-05 

Representative immunohistochemistry/confocal microscopy images of the CA1 region. Note the increase in size of astrocytic cell bodies in vanadium-treated group (bigger cell bodies and increased thickness of their processes) and the attenuation of astrocyte reactivity observed with the administration of ZA-II-05. Arrows indicate representative astrocytes and there was a significant difference (*p* < 0.05) between the vanadium, withdrawal, and ZA groups compared to the control and ZA-II-05-treated groups.

#### 2.5.5. Expression of IBA1 in Different Regions of the Brain Treated with Vanadium and ZA-II-05

Immunostaining showing microglia in the third ventricle, CA3, PFC, and Choroid plexus. In the vanadium group, note the increased number of activated microglia (soma and dendrites) and the bigger cell bodies compared to the control and ZA-II-05 treated groups which showed quiescence and recovery from activation. The level of microglial activation was significant (*p* < 0.05) across all the different brain regions. 

#### 2.5.6. Expression of Tumor Necrosis Factor (TNF) in Different Regions of the Brain Treated with Vanadium and ZA-II-05

Evaluation of tumor necrosis factor-alpha (TNF-α) in different brain regions: Purkinje cells (A), Cerebellar nuclei (B), Choroid plexus in the third ventricle (C), SN (D), Caudate putamen (E), Corpus callosum (F). Notice the increased expression of TNF-α in the groups treated with vanadium in the mentioned brain regions compared to the control and ZA-II-05 groups. The treated groups consistently downregulated TNF-α expression significantly (*p* < 0.05) in the substantia nigra, corpus callosum and caudate putamen. Interestingly, treatment with ZA-II-05 only upregulated the expression of TNF-α significantly (*p* < 0.05) in all the brain regions compared to the other groups.

## 3. Discussion

Vanadium toxicity is attributed to multiple mechanisms, but oxidative stress is considered a prominent factor. Animal studies have provided support for this hypothesis, as evidenced by the significant reduction in vanadium toxicity when antioxidants are administered either concurrently [21] or incorporated as coordination ligands [22]. As mentioned previously, disturbances in NMDAR activity have been linked to neurodegenerative disorders [23,24]. In this current study, we unravel new insights and novel findings that demonstrate the potential of ZA-II-05, a multi-target directed ligand (MTDL), in reversing vanadium-induced neurotoxicity. These findings suggest that ZA-II-05 could hold promise as a therapeutic approach for both vanadium neurotoxicity and neurodegenerative diseases. 

### 3.1. Calcium Dyshomeostasis following Treatment of CAD Cells with Vanadium

Multiple research studies have provided evidence linking calcium (Ca^2+^) homeostasis dysregulation to various neurodegenerative disorders [25,26]. In order to investigate the impact of disrupted Ca^2+^ ion balance on neuronal cells caused by vanadium salt (specifically sodium metavanadate), we employed Catecholaminergic a-differentiated (CAD) neuronal cells. This particular cell line possesses biochemical and morphological properties similar to primary dopaminergic neurons, making it a valuable tool for studying neuronal differentiation. The result supports our previous findings on the vulnerability of nerve processes to vanadium neurotoxicity compared to cell bodies [7]. This finding provides further evidence of disrupted calcium balance in the presence of vanadium exposure. Our previous study also observed a similar trend using calbindin, a calcium-binding protein [27], supporting the presence of calcium dyshomeostasis. The observed phenomenon (Figure 1) can be attributed to the perturbation of calcium signaling induced by vanadium, leading to the disturbance of regular neuronal communication and functioning. Additionally, it may result from the impairment of calcium buffering mechanisms due to excessive activation of calcium channels [28,29].

#### 3.1.1. ZA-II-05’s Binding Affinity Studies

In recent times, the concept of multi-target-directed ligands (MTDL) has been employed to create a range of molecules that target various biological factors associated with neurodegenerative diseases such as AD [30]. NMDA receptor antagonists, while primarily designed to target NMDA receptors, can sometimes exhibit binding affinity for other receptor types, such as dopamine, opioid, and sigma 2 receptors (Figure 2). This phenomenon is often referred to as “off-target” binding or cross-reactivity, and it occurs due to structural similarities and shared binding sites. 

The interaction between dopamine and NMDARs involves both direct and indirect mechanisms, which influence their function in different ways: Dopamine, through its D_1_-like receptors (D_1_ and D_5_), can positively modulate NMDAR function. When D_1_-like receptors are activated, the cAMP-PKA signaling pathway is activated, leading to the phosphorylation of NMDAR subunits, particularly the NR1 subunit [31]. This phosphorylation enhances the conductance and sensitivity of NMDARs to glutamate, resulting in increased synaptic plasticity and improved learning and memory [32,33]. 

It may also be possible that ZA-II-05 reduces vanadium excitotoxicity through its interaction with the sigma receptor; the exploration of sigma receptors as a potential treatment strategy for neurological disorders is a recent and noteworthy development [34]. Sigma-2 receptors are known to modulate calcium signaling, and NMDA receptors are one of the major sources of calcium influx in neurons. Therefore, it is possible that sigma-2 receptors influenced NMDAR-mediated calcium influx and, consequently, NMDAR function in this study [35,36,37]. The high binding affinity of ZA-II-05 in the studies suggests that it targeted sigma-2 receptor and modulated NMDA receptor function and calcium signaling. Combining sigma-2 receptor modulators with NMDA receptor-targeted drugs might offer a synergistic effect in certain neuropsychiatric disorders, providing enhanced therapeutic outcomes. 

NMDAR modulation by opioids could be indirect; opioid receptors, when activated, restrained gamma-aminobutyric acid (GABA) is released from interneurons, reducing inhibition on NMDAR activity [38]. This raises glutamate transmission, boosting NMDAR-mediated responses and excitatory synaptic transmission. This parallels dopamine-driven NMDAR phosphorylation effects. Dopamine and opioids are crucial in physiological and behavioral functions, shaping intricate synaptic communication and plasticity. Interaction between mu-opioid receptors (MOR) and NMDA receptors (NMDAR) adds complexity. MOR activation may alter NMDAR abundance and composition on cell membranes, suggesting linked functional modifications [38].

#### 3.1.2. Sub-Type Selectivity of ZA-II-05 for GluN2A and GluN2B 

Numerous studies have demonstrated that different subtypes of NMDA receptors produce distinct pharmacological and functional outcomes. Chazot et al. in 2004 [39] further emphasized the significance of targeting specific NR2 isoform(s) expressed in the receptor, i.e., GluN2A and GluN2B subunit-containing NMDA receptors, in conditions such as ischemic stroke and neurodegenerative diseases. Despite the lack of successful development in GluN2A and GluN2B-NMDA receptor antagonists thus far, researchers remain interested in these receptors and their significant potential for the application of GluN2A and GluN2B-NMDA receptor antagonists [40]. Our findings demonstrate that there was no significant difference in affinity for GluN1/GluN2A and GluN1/GluN2B in our novel compound (Figure 2), ZA-II-05. Both were within the same range of affinity. Selective antagonism of ZA-II-05 containing both GluN2A and GluN2B subtypes was probably responsible for ameliorating vanadium neurotoxicity in our study since both have been proven to be effective in curbing pathological roles of excitotoxicity neurological disorders [41]. Their unique characteristics and contributions provide more precise and tailored therapeutic interventions for various neurological conditions.

### 3.2. Acetylcholinesterase Activity

The current standard of care for Alzheimer’s disease (AD) patients is becoming increasingly focused on a combination of drugs that target both the cholinergic and glutamatergic systems [42]. This approach is founded on the idea that NMDA receptor antagonists could counteract neurodegeneration, while acetylcholinesterase inhibitors (AChEIs) could enhance memory and cognition by stimulating surviving cholinergic neurons. Acetylcholinesterase plays a crucial role as the primary enzyme responsible for the breakdown of the excitatory neurotransmitter acetylcholine into acetate and choline [43]. This enzymatic process takes place at neuronal synapses [44]. Consequently, an elevation in acetylcholinesterase activity leads to a depletion of acetylcholine [45,46]. Through a series of biochemical assays, we obtained a remarkable finding by measuring the activity of acetylcholinesterase (Figure 3). We propose that ZA-II-05 significantly (Table S1) inhibited increased acetylcholinesterase (AChE) activity through a combination of the following mechanisms: reduction in glutamate levels, which indirectly affects AChE activity [47]; regulation of calcium influx [48]; and suppression of neuroinflammatory processes [49]. An example of a channel blocker that reduces cholinesterase activity is the AD therapeutic, memantine [47,50].

### 3.3. Protective Effect of ZA-II-05 in Hippocampal Slices

In our in vitro studies, we focused on the hippocampus, a key region associated with memory in the brain (Figure 4). Previous research has shown that early life experiences, including stressful events, can affect the expression of NMDA receptors in the hippocampus [51,52] and may lead to a decrease in the number of granule cells [53]. ZA-II-05 demonstrated neuroprotection against vanadium-induced toxicity in the hippocampal slices (Figure 4). 

Excessive activation of glutamate receptors is known to contribute to the progressive degeneration of neurons. In our study, we observed a gradual loss of apical dendrites and cytotoxicity in the pyramidal cells of the CA1 and CA3 region (Figure 5). Based on these findings, we propose that these significant neuronal losses and hippocampal alterations are likely to contribute to memory impairment [54]. Our results in vivo demonstrate the vulnerability of the CA1 region to vanadium insults compared to the CA3 region. This susceptibility may be attributed to higher intrinsic superoxide levels and endogenous production of reactive oxygen species (ROS) within the CA1 region by vanadium. It has been reported that the mitochondrial permeability transition pore in the CA1 region is more sensitive to disturbances in calcium homeostasis, leading to increased ROS production [55]. 

Administration of ZA-II-05 exhibited neuroprotective effects against neuronal cell death in the pyramidal cells of the prefrontal cortex (PFC). It mitigated morphological alterations, suggesting an improvement in neuronal functionality (Figure 5). Interestingly, groups treated with ZA-II-05 only showed increased neuronal number and vitality, supporting ZA-II-05’s potential to protect neurons, probably through prevention of excitotoxicity via inhibition of excessive glutamate signaling [56]. The compound also possibly suppressed the release of pro-inflammatory cytokines, reducing neuroinflammation and its negative impact on neurons. Furthermore, ZA-II-05 probably modulated the activation of glial cells, such as microglia, to regulate neuroinflammatory responses and shield neurons from inflammatory harm. In summary, we suggest that ZA-II-05 employed a multifaceted approach to protect neurons by targeting various cellular processes involved in neuronal damage and supporting their survival.

### 3.4. Dendritic Morphology

Understanding the structural organization of these dendritic arbors becomes particularly important in the context of diseased conditions, as it contributes to the comprehension of altered brain network dynamics [57,58]. These improvements in dendritic length and arborization in pyramidal neurons in this study demonstrate a strong association with the amelioration of motor function and the attenuation of cognitive decline in the hippocampal and prefrontal neurons affected by vanadium-induced neurotoxicity [54]. Moreover, in this study, treatment with ZA-II-05 resulted in enhanced locomotion and improved motor coordination. We suggest that the observed enhancement in motor function can be attributed to the increased dendritic lengths and arborization in pyramidal neurons, similar to the findings reported in hippocampal and cortical neurons associated with the attenuation of cognitive decline [54]. This suggests a strong relationship between dendritic morphology and motor function improvement, as discussed earlier in relation to prefrontal neurons [58].

### 3.5. Glia Cells in Neurotoxicity Progression

Recent documentation has extensively established that disturbances in glial physiology can give rise to neurological disorders, including neurodegenerative diseases such as Parkinson’s disease, Alzheimer’s disease, and Huntington’s disease, as well as epilepsy, ischemic stroke, depression, autism, and glioma [59]. 

#### 3.5.1. Demyelination

There are three specific brain regions known for their high myelin content: the genu and splenium of the corpus callosum, and the arbor vitae [60,61]. Demyelination or hypomyelination has been documented as a consequence of vanadium exposure, either through lactation or direct intraperitoneal injection [7,14]. This preservation of myelin and neuronal structure integrity in the ZA-II-05 group (Figure 7) may be attributed to its scavenging of free radicals generated by vanadium. It is possible that ZA-II-05 prevents the penetration and actions of vanadium, although the exact mechanism requires further investigation. Notably, the demyelinating effect of vanadium and its mitigation through the use of ZA-II-05 is consistent with previous findings [27].

#### 3.5.2. Astrocytic Activation

**Figure 8:**
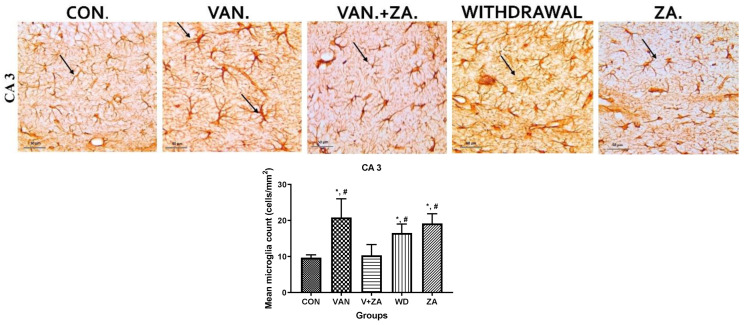
Shows the immunostaining of GFAP in the hippocampal CA3 region. It can be observed that the vanadium group displays increased activation of astrocytes, characterized by larger and thickened cell bodies with more extended and tortuous cytoplasmic processes, as well as increased thickness of their processes. Coadministration with ZA-II-05 attenuates the reactivity of astrocytes; they appear quiescent. The symbol * indicates a significant difference compared to the control group at *p* < 0.05, while the symbol # indicates a significant difference compared to the vanadium + ZA-II-05 (V+ZA) group at *p* < 0.05. Vanadium (100 mg/kg), and ZA-II-05 (100 mg/kg). The abbreviations used are as follows: CON for control, VAN for vanadium, ZA for ZA-II-05, and WD for withdrawal. Scale bar: 50 μm remains consistent across the rows.

Previous studies have reported vanadium-induced astrogliosis in the cerebellum and hippocampus of adult rats exposed to sodium metavanadate for 5 days, indicating a rapid response of astrocytes to this challenge [5]. In mice treated with vanadium, we observed astrocyte hypertrophy and enhanced GFAP immunostaining, specifically in the CA3 region of the hippocampus (Figure 8). The amelioration of these effects in the group treated with ZA-II-05 suggests its ability to modulate glutamate signaling and suppress pro-inflammatory signaling. These actions help reduce excessive astrocytic activation and mitigate the inflammatory response associated with astrogliosis induced by vanadium neurotoxicity. 

#### 3.5.3. Microglial Activation

**Figure 9:**
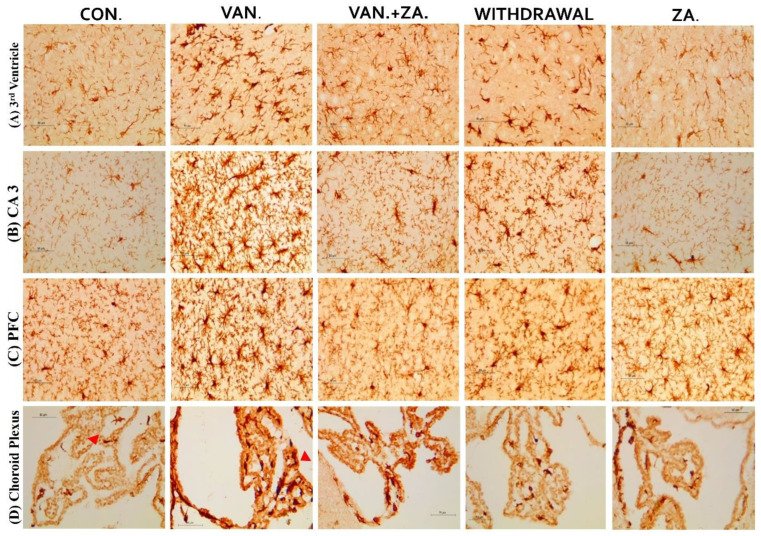

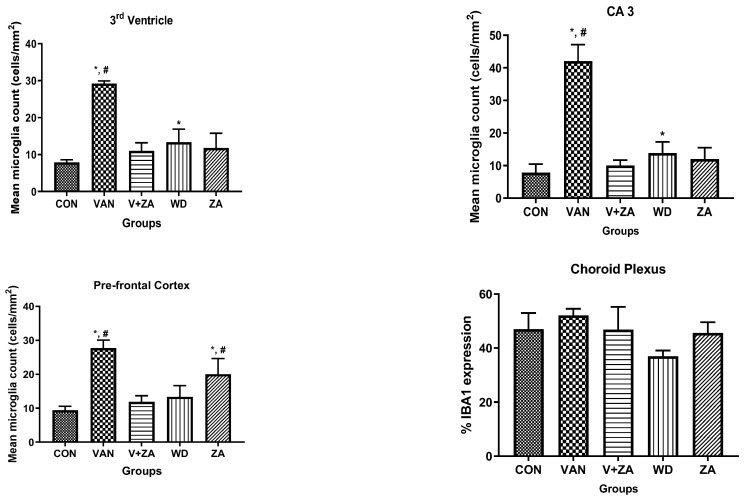
Panels show different brain regions (choroid plexus of the 3rd ventricle—(**A**), CA 3—(**B**), prefrontal cortex-PFC—(**C**), and choroid plexus of the lateral ventricles—(**D**)) were subjected to IBA-1 immunostaining after intermittent vanadium and ZA-II-05 treatment for 6 months. Microglial activation was observed; it was characterized by enlarged cell bodies with several short, thickened processes, in contrast to the matched controls that exhibited longer, finer branches. The vanadium-treated groups showed significant microglial activation in regions (**A**–**C**) compared to the control group. The ZA-II-05-treated group displayed improved microglial morphology relative to the vanadium-treated group. The number of IBA-1 positive cells in regions (**A**–**D**) was significantly increased in all vanadium-exposed groups compared to controls, while the ZA-II-05-treated group exhibited a reversal effect. In the statistical analysis, the symbol * indicates a significant difference compared to the control at *p* < 0.05, while # indicates a significant difference compared to the vanadium + ZA-II-05 (V+ZA) group at *p* < 0.05. Vanadium (100 mg/kg) and ZA-II-05 (100 mg/kg). The abbreviations used are as follows: CON for the control group, VAN for the vanadium-treated group, ZA for the ZA-II-05-treated group, and WD for the withdrawal group. Scale bar: 50 μm remains consistent across the rows.

Recent studies have focused on recognizing microglia as a potential therapeutic target for pharmacological interventions in different neurological disorders [62]. In the vanadium-treated group, we observed microglial activation in the CA 3 region of the hippocampus, choroid plexus, and the third ventricle of the brain. Environmental toxicants are known to readily trigger their activation [27]. The activated microglia displayed cellular hypertrophy, and their processes exhibited a bushy appearance. The degree of microglial activation was higher in the vanadium-treated group, indicating that vanadium-induced oxidative stress led to the production of free radicals (Figure 9). However, in the group treated with ZA-II-05, a noticeable decrease in microglial activation was observed, which was potentially attributed to its capacity to regulate glutamate signaling, thereby reducing excitotoxicity and inhibiting excessive calcium influx. 

#### 3.5.4. Tumour Necrosis Factor Expression

**Figure 10:**
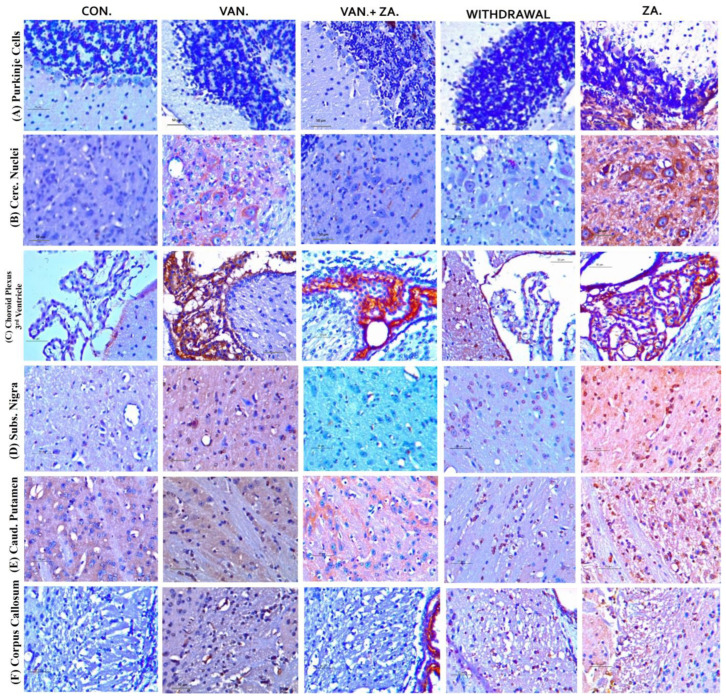

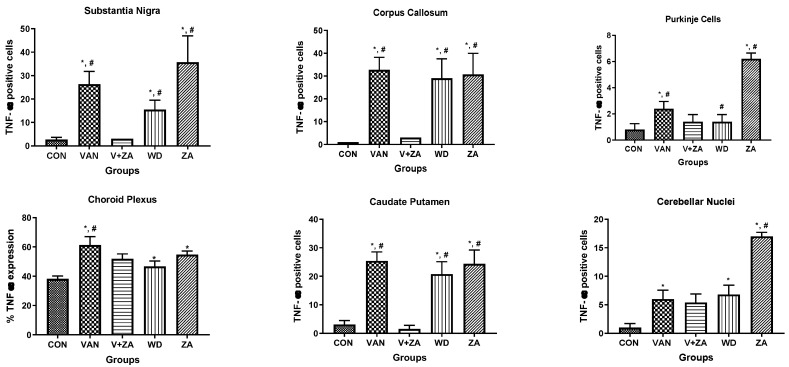
Immunohistochemical expression of brain tumor necrosis factor-alpha (TNF-α) in different brain regions, Purkinje cells (**A**), Cerebellar nuclei (**B**), Choroid plexus in the 3rd ventricle (**C**), SN (**D**), Caudate putamen (**E**), Corpus callosum (**F**) (×400). Control groups show negative TNF-α immunoreactivity. Vanadium-treated groups show cytoplasmic TNF-α immunoreactivity. Vanadium + ZA-II-05-treated groups show reduced cytoplasmic TNF-α expression compared to vanadium group. Groups treated with ZA-II-05 only show a significant increase in cytoplasmic TNF-α expression in the different brain regions. The symbol * is used to represent a significant difference compared to the control group at a significance level of *p* < 0.05, while the symbol # indicates a significant difference compared to vanadium + ZA-II-05 (V+ZA) group at the same significance level. Vanadium (100 mg/kg) and ZA-II-05 (100 mg/kg). CON, control; VAN, vanadium; ZA for ZA-II-05; WD; withdrawal. Scale bar = 50 μm (constant for all micrographs).

**Figure 11:**
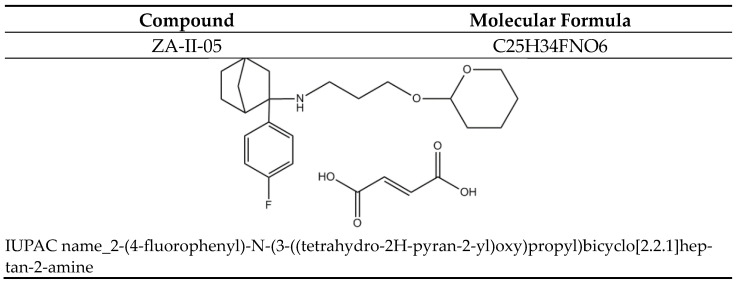
ZA-II-05.

Elevated levels of TNF alpha expression have been observed in the cerebrospinal fluid (CSF) of patients with various degenerative diseases such as AD, PD, MS, and stroke [63,64]. This may serve as a standalone prognostic indicator. In our study, treatment with ZA-II-05 resulted in a protective effect by downregulating TNF alpha expression in the treated group in different brain regions (Figure 10). This was likely a result of ZA-II-05′s (Figure 11) ability to interfere with intracellular signaling pathways involved in inflammation, such as the nuclear factor-kappa B (NF-κB) pathway. TNF-α expression is regulated by NF-κB [65], and NMDA receptor antagonists can inhibit NF-κB activation [66], leading to a decrease in TNF-α production. This is subject to further investigation, using ZA-II-05 antagonism. However, previous studies also showed that memantine inhibited the secretion of tumor necrosis factor-α and interleukin-1β triggered by ischemia–reperfusion in primary human brain microvascular endothelial cells [67]. These findings suggest that NMDA receptor antagonists may hold therapeutic potential in suppressing inflammation. Interestingly, administration of ZA-II-05 only led to elevated levels of TNF alpha expression in the different brain regions. This is not surprising, as previous studies have shown that administration of vitamin E, an antioxidant, in the absence of oxidative stress or a stressor, can be neurotoxic in mice brains [68]. The interaction between NMDA activity and TNF-alpha expression is intricate and can vary based on the specific brain region and dosage of administration.

## 4. Materials and Methods

### 4.1. Chemicals

Sodium metavanadate (NaO_3_V) (Sigma, St. Louis, MO, USA) was utilized in this study. Novel NMDA-Receptor antagonist, ZA-II-05, is a bicyclic aryl cyclohexylamine, designed and synthesized in our lab, fully active at 100 and 300 mg/kg (i.p.). Several tests established the safety of the compound at dosages envisioned in this study.

### 4.2. Neuronal Calcium Mobilization

We investigated the impact of vanadium on calcium homeostasis using CAD cells from a CNS catecholaminergic cell line called Cath a (Cath a differentiated) using methods described by [69]. To monitor changes in calcium levels, we prepared a 2 mM stock solution of Calcium Green™-1AM probe, which was stored at −20 °C. On the day of the imaging experiments, the probe stock was heated to room temperature and diluted to a 1 µM solution in HEPES physiological buffer. The cells were plated in flat-bottom micro dishes and incubated with the Calcium Green solution for 40 min. Subsequently, the cells were washed and maintained in HEPES buffer prior to imaging. All experimental reagents were dissolved in the HEPES buffer, and each treatment involved specific incubation and imaging periods. The imaging was performed using a Zeiss LSM 850 microscope equipped with an Airyscan system, utilizing a laser with specific settings. The fluorescence images were captured using a size 63 oil lens, and the time series intervals and frame duration were adjusted accordingly. The figures and fluorescence intensities were analyzed using the Zeiss LSM 850 with an Airyscan microscope. Overall, this experimental setup allowed us to examine the effects of vanadium on calcium dynamics in the CAD cells.

### 4.3. Pharmacological Characterization of ZA-II-05

#### 4.3.1. Radioligand Binding Assay

ZA-II-05’s in vitro binding affinities (Ki) at the PCP site within the NMDAR channel and other neurotransmitters were assessed using competitive radioligand binding studies with [3 H]-MK-801 at the following receptors 5-HT2A, Dopamine 1 (D1), Dopamine 1(D2), Dopamine Transporter (DAT), Opioid receptors (KOR), µ-opioid receptors (MOR), Norepinephrine Transporter (NET) and Sigma receptor following the established protocol by Reynolds and Sharma [70,71]. The binding profile data were used to calculate the Ki values, which represent the binding affinities. The experiments were conducted in duplicate and repeated a minimum of three to five times.

#### 4.3.2. Subtype Ligand Binding Assay

The expression of NMDA receptor subunit clones in ZA-II-05, following their transfection in HEK 293 cells, was determined according to the methods described by [72]. Briefly, HEK 293 cells were transferred to multiple flasks and cultured until they reached 50% confluence. The cell-culture media was replaced with fresh media and CO_2_ levels were adjusted prior to transfection. Samples containing plasmid DNA and buffer were prepared. Calcium chloride was added to the plasmid DNA solution and mixed, and the resulting solution was then added to the cell flask. Glycerol shock was performed to enhance transfection efficiency, followed by media replacement. Transfected cells were harvested, homogenized, and centrifuged to obtain cell pellets.

### 4.4. Biochemical Tests

Biochemical changes were assessed in all the treatment groups using a total of 20 whole mouse brains from the aforementioned five groups. Twenty-four hours after the final treatment, each subgroup of animals (n = 4) was sacrificed for analysis. The determination of nitrite (NO) content followed the methods described by [73]. Glutathione-S transferase (GST) levels were estimated using the procedures outlined by [74]. Vitamin C levels were determined according to the methodology described by [75]. The measurement of H_2_O_2_ generation was conducted according to the methods established by [76]. Non-protein thiol (NPT) and total thiol (PT) content were estimated using the technique developed by [77]. Acetylcholinesterase (AChE) activity was measured as described by [77].

### 4.5. Measurement of Protective Effect of ZA-II-05 In Vitro in Hippocampal Slices

ZA-II-05 was evaluated for its in vitro neuroprotective effects using the in vitro hippocampal slice culture assay at several concentrations (10 μM, 20 μM, 50 μM and 100 μM) as described by [78]. Neurotoxicity was induced by treating organotypic hippocampal slice cultures obtained from Sprague Dawley rat pups (PND 10-11) with vanadium at 300 μM concentration. Cell death was quantified by measuring the uptake of 2 μM propidium iodide, and the intensity of propidium iodide fluorescence was measured using EVOS FL digital inverted microscope with Texas Red filter at 80% transmittance with 1.0 s exposure and 4× magnification. Statistical analysis was performed to determine the percentage of significant differences between groups for propidium iodide uptake using one-way ANOVA.

### 4.6. Experimental Design and In Vivo Drug Administration

The mice utilized in this study were sourced from the animal facility of the Department of Veterinary Antaomy at the University of Ibadan, Nigeria. Five pregnant mice were included, each giving birth to eight pups (n = 8 mice per dam). The mice were randomly assigned to five different test groups. They were housed in the animal house of the Neuroscience Unit, Department of Veterinary Anatomy, University of Ibadan, and provided with unrestricted access to food and water. Ethical approval for the study was obtained from the Animal Ethical Committee of the University of Ibadan, with the assigned ethical code number: UI-ACUREC/17/0035.

Group 1: (Control) mice (n = 8) were injected with sterile water (vehicle) every 72 h intraperitoneally (i.p) for a duration of six (6) months. Body weight was monitored daily using an electronic weighing balance (KERN EW, Germany) across all the groups.

Group (2): (Vanadium) mice (n = 8) were injected with sodium metavanadate (3 mg/kg) every 72 h intraperitoneally (i.p) for six months to induce neurodegeneration.

Groups (3): (Vanadium + ZA-II-05) mice (n = 8) were treated with both sodium metavanadate (3 mg/kg) and ZA-II-05 (100 mg/kg) i.p every 72 h for six months to test the ameliorative effect of the compound on vanadium-induced neurotoxicity.

Groups (4): (Withdrawal) mice (n = 8) were treated with sodium metavanadate (3 mg/kg) i.p daily for five months and replaced with sterile water to the sixth month to examine the withdrawal effects of vanadium administration.

Groups (5): (ZA-II-05) mice (n = 8) were treated with ZA-II-05 only (100 mg/kg) i.p every 72 h for one month, (from the fifth to the sixth month) to test the effect of the compound in the absence of vanadium administration.

#### Immunohistochemistry

The brains were prepared and embedded in paraffin wax, and 4 μm-thick sections were obtained. Immunohistochemical procedures were performed following the methods described by [79]. To block the sections, 2% milk was used for a duration of 1 h. Subsequently, the sections were probed with the respective antibodies (listed in Table 1) overnight. The antibodies used included anti-Glial Fibrillary Acidic Protein Rabbit Polyclonal (GFAP) antibody, which was used to assess astrocyte morphology (ab4648; 1:800, Abcam, Cambridge, UK); anti-ionized calcium binding adapter molecule-1 (IBA-1) rabbit polyclonal antibody, which was used to examine microglia morphology (1:1000); anti-Myelin Basic Protein (MBP) rabbit polyclonal antibody, which was used to evaluate myelination (ab216590; 1:800, Abcam, Cambridge, UK); and anti-Tumour Necrosis Factor (TNF) monoclonal mouse antibody (ab212899; 1:200, Abcam, Cambridge, UK) and Neuronal nuclear antigen (NeuN) rabbit monoclonal antibody (ab104224; 1:500, Abcam, Cambridge, UK) for a duration of 16 h at 4 °C in the appropriate biotinylated secondary antibodies (diluted 1:200; all purchased from Abcam, Cambridge, UK. Immunoreactive cells were quantified and analyzed using ImageJ software version 1.52f for Windows.

### 4.7. Golgi Staining

For the Modified Golgi stain, a subset of three mouse brains per group was selected according to a protocol described in a previous study [58]. This staining method was utilized to examine the dendritic arborization of neurons in the cornus ammonis (CA) 1 region of the hippocampus, as well as the cerebral and cerebellar regions. Neurons that exhibited clear silver impregnation and well-defined cell bodies and processes were chosen for morphological assessment based on qualitative analysis.

### 4.8. Statistical Analysis

The statistical analyses were conducted using Graphpad Prism version 9.5.1 (733) for Windows, Graphpad Software Inc. Data, presented as mean ± standard deviation (SD), were obtained from a minimum of 5 samples per group. One-way Analysis of Variance (ANOVA) was utilized for group comparisons. Tukey’s test was employed to compare the means of individual groups. A significance level of *p* ≤ 0.05 was considered statistically significant.

## 5. Conclusions

In conclusion, continued exposure to heavy metals, such as vanadium, remains a persistent environmental and health concern, elevating the risk of neurodegenerative disorders. Vanadium’s ability to breach the blood–brain barrier adds to the complexity of cognitive impairments and neuronal damage associated with its toxicity. Importantly, the administration of ZA-II-05 preserved cellular integrity in several anatomical regions, indicating its potential as a countermeasure against vanadium-induced neurotoxicity. These findings highlight the promise of ZA-II-05 as a candidate for further research and development in the quest to protect against the neurological hazards posed by heavy metal exposure, particularly vanadium.

## Figures and Tables

**Table 1:** Receptors and their sources screened for ZA-II-05.

Receptor	[^3^H] Ligand	Species	Source
5-HT_2A_	Ketanserin	Human	Cloned
Dopamine 1 (D1)	SCH23390	Human	Cloned
Dopamine 1(D2)	N-Methylspiperone	Human	Cloned
Dopamine Transporter (DAT)	WIN35428	Human	Cloned
*κ*-opioid receptors (KOR)	U69593 (2007-07-27)	Rat	Cloned
*µ*-opioid receptors (MOR)	DAMGO (2007-07-27)	Human	Cloned
Norepinephrine Transporter (NET)	Nisoxetine	Human	Cloned
Serotonin Transporter (SERT)	Citalopram	Human	Cloned
Sigma_1_	Pentazocine (+)	Rat	Brain
Sigma_2_	DTG	Rat	PC12

## Data Availability

Data are contained within the article and supplementary materials.

## Supplementary Materials

The supporting information can be downloaded at: https://www.mdpi.com/article/10.3390/ijms242316710/s1.
