# Supplementary material for: Attenuation of Vanadium-Induced Neurotoxicity in Rat Hippocampal Slices (In Vitro) and Mice (In Vivo) by ZA-II-05, a Novel NMDA-Receptor Antagonist
Source: Int J Mol Sci. 2023 Nov 24;24(23):16710. doi: 10.3390/ijms242316710 (PMC10706475; doi:10.3390/ijms242316710)
Supplement: Supplementary file 1 [file ijms-24-16710-s001.zip › ijms-2672760-supplementary.pdf]

Supplementary Materials

Table S1. Showing non-enzymatic antioxidant content, Hydrogen perioxide-H_2O_2, Nitric oxide and Acetylcholinesterase activities in the different treatment groups. Superscript (x) shows significance difference ($P < 0.05$) of vanadium exposure relative to control and ZA-II-05 treated group. NPT (Non-protein thiol).

Parameter	Groups				
	**CONTROL**	**VANADIUM**	**VAN.+ZA.**	**WITHDRAWAL**	**ZA-II-05**
**AchE** (mmole of substrate/min/mg/protein)	28.00±2.62	346.31±32.57^x	44.58±4.54	259.74±13.34	20.00±6.49
**THIOL** (nmoles/mg/protein)	28.14±2.10	22.08±1.01	25.06±4.68	23.82±1.10	28.62±6.72
**NPT** (nmoles/mg protein)	31.82±5.88	23.45±2.28	29.06±3.80	32.24±5.91	26.98±5.89
**VIT. C** (nmoles/mg protein)	0.18±0.02	0.162±0.01	0.20±0.04^x	0.16±0.02	0.17±0.02
**H_2O_2** *(mmole* H_2O_2 */min/mg/protein)*	43.73±2.40	46.02±0.29	39.30±4.17	38.89±2.67	39.15±3.49
**NO** (μmole/mg protein)	0.32±0.02	0.38±0.04	0.32±0.03^x	0.32±0.03	0.34±0.03

Profile of brain antioxidant enzymes, vitamin C, total thiol (PT), and non-protein thiol (NPT) levels in mice exposed to vanadium and ZA-II-05.
